# Nationwide in‐hospital mortality rate following rectal resection for rectal cancer according to annual hospital volume in Germany

**DOI:** 10.1002/bjs5.50254

**Published:** 2020-01-10

**Authors:** J. Diers, J. Wagner, P. Baum, S. Lichthardt, C. Kastner, N. Matthes, H. Matthes, C.‐T. Germer, S. Löb, A. Wiegering

**Affiliations:** ^1^ Department of General, Visceral, Transplant, Vascular and Paediatric Surgery University Hospital, University of Würzburg Würzburg Germany; ^2^ Comprehensive Cancer Centre Mainfranken University of Würzburg Medical Centre Würzburg Germany; ^3^ Department of Biochemistry and Molecular Biology University of Würzburg Würzburg Germany; ^4^ Gemeinschaftskrankenhaus Havelhöhe Berlin Germany

## Abstract

**Background:**

The impact of hospital volume after rectal cancer surgery is seldom investigated. This study aimed to analyse the impact of annual rectal cancer surgery cases per hospital on postoperative mortality and failure to rescue.

**Methods:**

All patients diagnosed with rectal cancer and who had a rectal resection procedure code from 2012 to 2015 were identified from nationwide administrative hospital data. Hospitals were grouped into five quintiles according to caseload. The absolute number of patients, postoperative deaths and failure to rescue (defined as in‐hospital mortality after a documented postoperative complication) for severe postoperative complications were determined.

**Results:**

Some 64 349 patients were identified. The overall in‐house mortality rate was 3·9 per cent. The crude in‐hospital mortality rate ranged from 5·3 per cent in very low‐volume hospitals to 2·6 per cent in very high‐volume centres, with a distinct trend between volume categories (*P* < 0·001). In multivariable logistic regression analysis using hospital volume as random effect, very high‐volume hospitals (53 interventions/year) had a risk‐adjusted odds ratio of 0·58 (95 per cent c.i. 0·47 to 0·73), compared with the baseline in‐house mortality rate in very low‐volume hospitals (6 interventions per year) (*P* < 0·001). The overall postoperative complication rate was comparable between different volume quintiles, but failure to rescue decreased significantly with increasing caseload (15·6 per cent after pulmonary embolism in the highest volume quintile *versus* 38 per cent in the lowest quintile; *P* = 0·010).

**Conclusion:**

Patients who had rectal cancer surgery in high‐volume hospitals showed better outcomes and reduced failure to rescue rates for severe complications than those treated in low‐volume hospitals.

## Graphical abstract

In‐hospital mortality after rectal cancer surgery is strongly correlated with annual hospital caseload. This is the result of an increased failure‐to‐rescue rate in the case of postoperative complications in low‐volume hospitals rather than the result of an increased overall rate of complications.

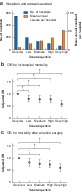

Rectal cancer surgery and hospital volume

## Abstract (es)

**Antecedentes:**

El impacto del volumen hospitalario en los resultados de la cirugía del cáncer de recto ha sido poco investigado. Este estudio tuvo como objetivo analizar el impacto de los casos anuales de cirugía de cáncer de recto por hospital en la mortalidad postoperatoria (*postoperative mortality*, POM) y el fracaso en el rescate (*failure to rescue*, FtR).

**Métodos:**

Todos los casos de pacientes hospitalizados con un diagnóstico de cáncer de recto y un código de procedimiento de resección rectal, tratados de 2012 a 2015, se identificaron a partir de datos hospitalarios administrativos a nivel nacional. Los hospitales se agruparon en cinco quintiles según el volumen de casos. Se determinó el número absoluto de pacientes, la POM y el FtR por complicaciones postoperatorias graves. El FtR se definió como la mortalidad hospitalaria después de una complicación postoperatoria documentada.

**Resultados:**

Se identificaron 64.349 casos entre 2012 y 2015. La tasa de mortalidad hospitalaria global fue del 3,89% (*n* = 2.506). Las tasas brutas de mortalidad hospitalaria variaron de 5,34% (*n* = 687) en hospitales de muy bajo volumen a 2,63% (*n* = 337) en centros de muy alto volumen, con una tendencia distinta entre las categorías de centros (*P* < 0,001).

En el análisis de regresión logística multivariante utilizando el volumen hospitalario como efecto aleatorio, los hospitales de muy alto volumen (53 intervenciones/año) tenían una razón de oportunidades (*odds ratio*, OR) ajustada por riesgo de 0,58 (i.c. del 95%: 0,47‐0,73) en comparación con la tasa basal de mortalidad hospitalaria en hospitales de muy bajo volumen (6 intervenciones/año) (*P* < 0,001). La tasa global de complicaciones postoperatorias fue comparable entre los diferentes quintiles de volumen, pero el FtR disminuyó significativamente con el aumento del volumen de casos (15,63% FtR tras una embolia pulmonar en el quintil más alto *versus* 38,4% en el hospital del quintil más bajo, *P* = 0,01).

**Conclusión:**

Los pacientes sometidos a cirugía de cáncer de recto en hospitales de gran volumen presentaron mejores resultados y una disminución de las tasas de fracaso en el rescate por complicaciones graves en comparación con los pacientes tratados en hospitales de bajo volumen.

## Introduction

With an incidence of one million new cases and half a million deaths per year, colorectal cancer is the most common malignancy of the gastrointestinal tract worldwide^1^. Approximately 30 per cent of these tumours are located in the rectum. To treat colorectal cancer more effectively, the concept of multimodal therapy is well established; this includes neoadjuvant radiochemotherapy, surgery with mesorectal excision and adjuvant chemotherapy. This multimodal approach has led to a reduction in the rate of local tumour recurrence and a substantial improvement in the long‐term survival of patients with rectal cancer^2^, ^3^, ^4^, ^5^. However, it remains unclear whether hospital volume, surgeon volume or the expertise of the individual surgeon also contributes to the effect on short‐ and long‐term outcomes.

A Cochrane review^6^ suggested that both hospital volume and surgeon specialization significantly influence long‐term survival, but that short‐term (30‐day) mortality depends only on surgeon specialization grade. However, the data included in the review^6^ were acquired over a long time interval and most patients did not have multimodal therapy, which might have affected the outcome. Furthermore, the definition of hospital volume was heterogeneous and the number of pooled patients was relatively low. A systematic review and meta‐analysis^7^ of 45 275 patients with rectal cancer treated within a multimodal setting showed reduced postoperative mortality for those treated in high‐volume hospitals.

The aim of this study was to analyse in‐hospital mortality after rectal cancer resection according to annual hospital volume in Germany. Nationwide billing data from 2012 to 2015 for patients with a diagnosis of either rectal (C20) or rectosigmoidal cancer (C19) and a simultaneous therapy code for rectal or rectosigmoidal resection were included and analysed (5484/5, 54556/7, 54581/5). The primary endpoint was the in‐hospital mortality rate, and the secondary endpoint was ‘failure to rescue’ in patients with postoperative complications.

## Methods

A register‐based, retrospective cohort study based on individual inpatient data from nationwide German diagnosis‐related groups (DRG) statistics was conducted. Data on all German inpatients with a DRG code for cancer of the rectosigmoid or rectum as the main diagnosis who had resection between 1 January 2012 and 31 December 2015 were included. Patients were divided into five cohorts according to the total caseload of rectal resection in their hospital during this period^8^.

### Case definition and hospital volume

All patients with the DRG code C19 for rectosigmoid cancer or C20 for rectal cancer as the principal diagnosis, and an associated procedure code for rectal or rectosigmoid resection (5484/5, 54556/7, 54581/5) were included in the study. Procedures were considered hierarchically for each patient. More radical procedures were defined as the principal intervention to avoid double‐counting interventions done in the same patient.

Hospitals were ranked according to their rectal cancer resection volume, based on pooled 2012–2015 data. Five volume categories with an approximately equal number of patients were generated. Hospital volume was also examined as a continuous variable.

### Data

With the exception of psychiatric patients, acute hospitals in Germany are obliged by law to report DRG and procedure coding data for all inpatients to the Federal Statistical Office, and to the Länder offices for statistical purposes. The data also serve as the basis for hospital reimbursement. DRG data were accessed by controlled remote data analysis via the Research Data Centre of the Federal Statistical Office. For legal reasons and because of data protection regulations, direct access to the raw data is not possible. Data provided by the Research Data Centre include primary and secondary diagnoses DRG codes, procedure codes, sex, patient age and length of hospital stay (LOS). The German adaptation of ICD‐10‐GM codes and relevant versions (2012–2015) of the German procedure codes were used for patient identification and data analysis^9^. The analysis was restricted to complete data records. If there were duplicate data, one data set was chosen at random for further analysis.

Data obtained from records included: demographics, type of surgical procedure, location of the tumour, setting (elective/emergency), mechanical ventilation for 48 h or more, massive transfusion, co‐morbidity, LOS and complications.

### Co‐morbidity and potential confounders

To account for differences in the range of co‐morbidity between hospital volume quintiles, the co‐morbidity score for each patient was determined, as proposed by Stausberg and Hagn^10^. This score is based on the structure of the ICD‐10 groups and has been validated in a large cohort of German patients; it outperformed other indexes commonly used to control for confounding by co‐morbidity, such as the Charlson and Elixhauser co‐morbidity indexes^10^. Data on other potential confounders, such as sex, age or emergency procedure, were also considered and included in the analysis (*Table* *S1*, supporting information).

### Outcome measures

The main study outcome was in‐hospital mortality (death while an inpatient regardless of LOS). The secondary aim was to investigate trends in failure to rescue, defined as in‐hospital death after diagnosis of a postoperative complication.

### Statistical analysis

The raw data were screened for missing values and checked for plausibility. The continuous variable of age was recoded as a categorical dummy variable with three age categories: 59 years or less, 60–74 years and 75 years or above. These cut‐offs ensured similar sizes for the second and third age groups, and confined patients with a presumably higher incidence of genetic aberrations leading to early‐onset cancer to one age group. Patient characteristics were analysed descriptively for each year and as a function of hospital volume quintiles. Differences between subgroups were assessed using χ^2^ tests where appropriate. Temporal trends and trends across volume categories were assessed by means of a non‐parametric test for trend, as described by Cuzick^11^. Second, crude odds ratios (ORs) between the main dependent variable (in‐hospital mortality) and the main independent variable (hospital volume quintile) were calculated using the pooled data. In addition, crude ORs between the secondary independent variables (listed below), the main independent variable and the outcome of interest were determined to identify potential confounders. The possibility of important effect modification was assessed by means of the Mantel–Haenszel method, adjusting for each potential confounder. The correlation between each pair of variables was determined to detect multicollinearity.

The effect of hospital volume on in‐hospital mortality was evaluated using a multivariable logistic regression model, which included hospital volume as a random effect to account for clustering of patients in different institutions. The multivariable model was adjusted for known confounding effects of sex, age, emergency procedure and co‐morbidity. Models were fitted with the number of patients per hospital as a continuous variable and hospital volume quintile as a linear variable. Likelihood ratio tests were used to assess the fit of models and to evaluate the presence of linear trends.

The accuracy of the random‐effects estimators of the multivariable regression models was checked by refitting the models for different numbers of quadrature points and subsequent comparison of the values of the estimators. A maximum relative difference of 10^−4^ or less between the different quadrature points was considered acceptable.

Where appropriate, 95 per cent c.i. and *P* values were determined. *P* ≤ 0·050 was considered statistically significant. All statistical calculations were done with Stata® version 14.2 (StataCorp, College Station, Texas, USA).

## Results

A total of 64 411 patients with a diagnosis of either rectosigmoidal or rectal cancer (ICD codes C19 and C20) reported to the German Federal Statistical Office, who subsequently had rectal surgery (procedure codes 5484 and 5485, with their relevant subgroups, or 54556/7 and 54581/5) between 1 January 2012 and 31 December 2015, were included. Sixty‐one patients were excluded from further analysis owing to duplication. One patient had missing data. Consequently, missing or duplicated data occurred at a rate of 0·1 per cent (62 of 64 411), resulting in a final data set of 64 349 patients for further analysis.

Some 23 999 (37·3 per cent) of the patients were women, and the median age was 70 years. The nationwide mean annual number of patients with rectal cancer treated surgically was 16 087. Emergency procedures accounted for 18·4 per cent (11 826) of all operations during the 4‐year period. A majority of patients (57 034, 88·6 per cent) had rectal cancer (DRG code C20), and the remaining 7315 were treated for rectosigmoid cancer (DRG code C19).

The most frequent surgical procedures were sphincter‐preserving anterior resection (15 380, 23·9 per cent) and sphincter‐preserving low anterior resection (28 888, 44·9 per cent) (*Table* 1). Non‐sphincter‐sparing rectal resection was performed in 13 518 patients, accounting for 21·0 per cent of all operations during the 4‐year interval. The resection was performed laparoscopically in 18 867 patients (29·3 per cent). No temporal trends in the total number of patients, or in patient age or co‐morbidity were observed from 2012 to 2015. However, mean LOS after rectal cancer resection decreased steadily (21·6 days in 2012 *versus* 19·9 days in 2015; *P* < 0·001).

**Table 1:** Characteristics of patients undergoing rectal resection for rectal cancer in 2012–2015, according to hospital volume quintile

	Hospital volume quintile					
	Very low	Low	Medium	High	Very high	*P* ‡
**No. of hospitals**	550	197	137	101	61	
**Total no. of patients**	12 864	12 738	12 989	12 916	12 842	
**In‐hospital deaths**	687 (5·3)	562 (4·4)	477 (3·7)	443 (3·4)	337 (2·6)	< 0·001§
**No. of patients over 4‐year period** *	23·4(14·9)	64·7(9·0)	94·8(8·6)	127·9(12·0)	210·5(64·3)	
**Annual volume per hospital** *	5·8	16·2	23·7	32·0	52·6	
**Age (years)** *	70·3(11·2)	69·1(11·2)	68·2(11·5)	67·5(11·5)	66·6(11·7)	< 0·001§
≤ 59†	2415 (16·3)	2660 (17·9)	2995 (20·2)	3233 (21·8)	3523 (23·8)	
60–74†	5253 (18·6)	5582 (19·8)	5701 (20·2)	5816 (20·6)	5826 (20·7)	< 0·001
≥ 75†	5196 (24·3)	4496 (21·1)	4293 (20·1)	3867 (18·1)	3493 (16·4)	
**No. of women**	4991 (38·8)	4813 (37·8)	4929 (37·9)	4617 (35·7)	4649 (36·2)	< 0·001§
**Co‐morbidity score** *	102·3(5·2)	102·1(5·3)	101·7(5·0)	101·6(4·9)	101·5(4·9)	< 0·001§
**Length of hospital stay (days)** *	21·7(14·6)	21·6(15·2)	20·5(14·5)	20·6(15·8)	19·7(15·0)	< 0·001§
**Cancer location**						
Rectosigmoid	2281	1514	1201	1197	1122	
Mortality	129 (5·7)	78 (5·2)	56 (4·7)	43 (3·6)	31 (2·8)	0·001
Rectum	10 583	11 224	11 788	11 719	11 720	
Mortality	558 (5·3)	484 (4·3)	421 (3·6)	400 (3·4)	306 (2·6)	< 0·001
**Type of surgery**						
Non‐sphincter‐preserving rectal resection	2548	2686	2804	2808	2672	
Mortality	134 (5·3)	134 (5·0)	106 (3·8)	116 (4·1)	76 (2·8)	< 0·001
Sphincter‐preserving resection and perianal anastomosis	850	1020	980	821	972	
Mortality	36 (4·2)	28 (2·7)	19 (1·9)	10 (1·2)	14 (1·4)	< 0·001
Sphincter‐preserving low anterior resection	5159	5383	5914	6081	6351	
Mortality	245 (4·7)	214 (4·0)	202 (3·4)	172 (2·8)	155 (2·4)	< 0·001
Sphincter‐preserving anterior resection	3670	3269	2937	2902	2602	
Mortality	210 (5·7)	149 (4·6)	125 (4·3)	122 (4·2)	73 (2·8)	< 0·001
Other resection (sigmoid/left)	497	261	258	237	197	
Mortality	54 (10·9)	31 (11·9)	19 (7·4)	18 (7·6)	19 (9·6)	0·390
Tubular/segmental resection	140	119	96	67	48	
Mortality	n.s.	n.s.	6 (6·3)	n.s.	n.s.	–
Sphincter‐preserving (low anterior) resection	9679	9672	9831	9804	9925	
Mortality	491 (5·1)	391 (4·0)	346 (3·5)	304 (3·1)	242 (2·4)	< 0·001
Any laparoscopic resection	3252	3672	4026	4202	3715	
Mortality	99 (3·0)	91 (2·5)	84 (2·1)	66 (1·6)	61 (1·6)	< 0·001
Laparoscopic sphincter‐preserving low anterior resection	2635	2985	3243	3415	3097	
Mortality	65 (2·5)	63 (2·1)	60 (1·9)	47 (1·4)	48 (1·5)	0·017

Values in parentheses are percentages of total in the relevant quintile unless indicated otherwise;

* values are mean(s.d.);

† values in parentheses are percentage of total in that age group. n.s., Not stated owing to German data protection legislation.

‡ χ^2^ test for difference between subgroups, except

§ non‐parametric test for trend.

The nationwide overall in‐hospital mortality rate for rectal cancer surgery was 3·9 per cent (2506 of 64 349) (*Table* 1). The mortality rate increased with increasing age, varying from 0·8 per cent (126 of 14 826) in patients aged 59 years or less, to 2·7 per cent (766 of 28 178) in patients aged 60–74 years and 7·6 per cent (1614 of 21 345) in patients aged 75 years or above. The in‐hospital mortality rate was higher for men than for women: 4·0 per cent (1634 of 40 350) *versus* 3·6 per cent (872 of 23 999) respectively. The in‐hospital mortality rate was generally higher in patients with rectosigmoid carcinoma (337 of 7315, 4·6 per cent) compared with that in patients with rectal cancer (2169 of 57 034, 3·8 per cent). In general, laparoscopic resection was associated with decreased mortality (2·1 per cent (401 of 18 867) *versus* 3·9 per cent for overall in‐hospital mortality; *P* < 0·001). For sphincter‐preserving low anterior resection, the mortality rate was lower in patients who had a laparoscopic resection than in those having open surgery (1·8 per cent (283 of 15 375) *versus* 4·5 per cent (1516 of 34 006) respectively; *P* < 0·001).

Emergency procedures, mechanical ventilation for 48 h or more, and massive transfusion were all associated with a significantly higher mortality rate (emergency procedure: 7·2 per cent (856 of 11 826) *versus* 3·1 per cent (1650 of 52 523) for non‐emergency procedures; mechanical ventilation: 34·1 per cent (1021 of 2997) *versus* 2·4 per cent (1485 of 61 352) for no ventilation; transfusion: 22·4 per cent (786 of 3509) *versus* 2·8 per cent (1720 of 60 840) for no transfusion; all *P* < 0·001). Relaparotomy, including adhesiolysis and surgical decompression of the gastrointestinal tract as indicative of postoperative complications, was also associated with increased in‐hospital mortality (15·9 per cent (648 of 4078) *versus* 3·1 per cent (1858 of 60 271) for no relaparotomy; *P* < 0·001). Anastomotic leak, reported in 11·8 per cent of all procedures with an anastomosis (5998 of 50 831), showed a highly significant association with in‐hospital death (8·2 per cent (492 of 5998) *versus* 3·5 per cent (2014 of 58 351) in those with no anastomosis; *P* < 0·001), as did the occurrence of postoperative peritonitis/sepsis (18·4 per cent (1200 of 6530) *versus* 2·3 per cent (1306 of 57 819) in those with no sepsis; *P* < 0·001).

### Trends across hospital volume categories

**Figure 1:**
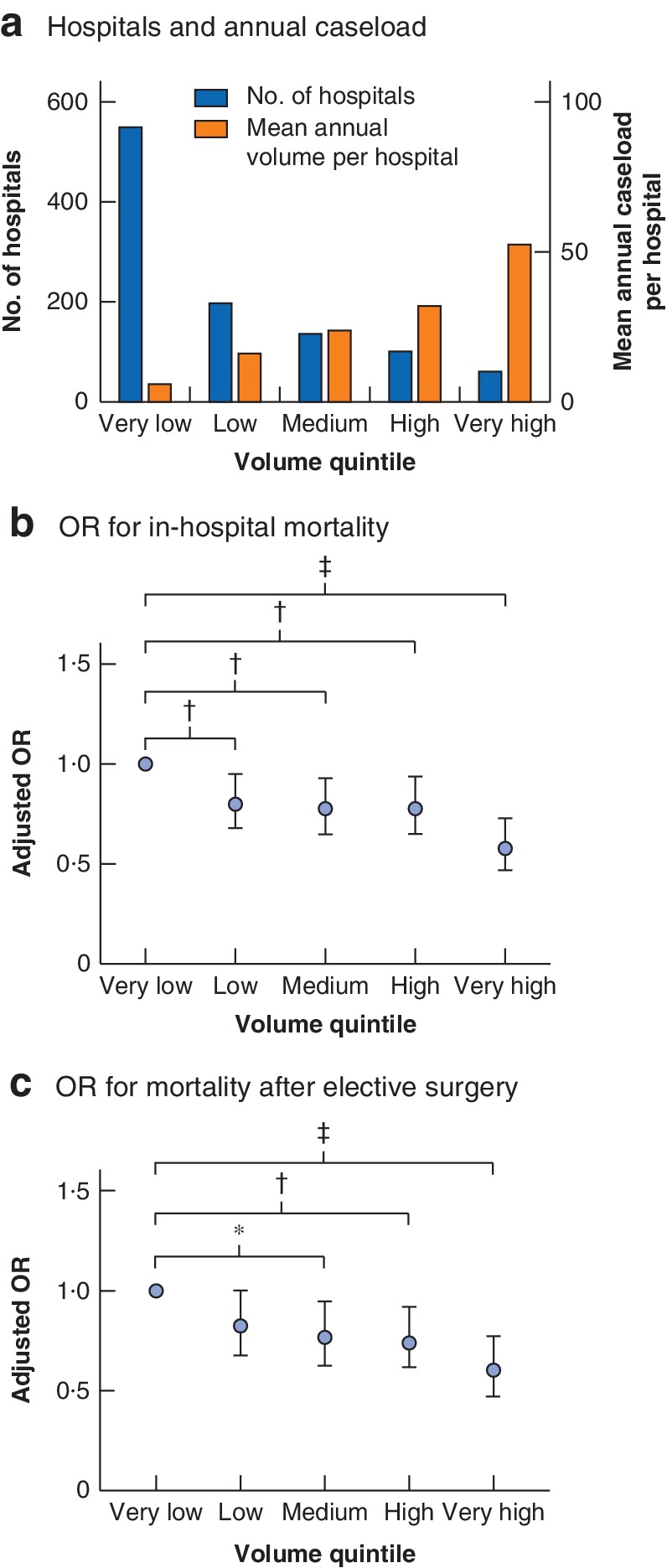
Hospitals, hospital caseload and mortality risk according to hospital volume quintiles **a** Number of hospitals and mean annual caseload per hospital per year. **b** Risk‐adjusted odds ratios (ORs) with 95 per cent c.i. for in‐hospital mortality. **c** Risk‐adjusted ORs and 95 per cent c.i. for in‐hospital mortality following elective surgery. *P < 0·050, †*P* ≤ 0·010, ‡*P* < 0·001 (logistic regression analysis).

The 1046 hospitals were grouped into five equal caseload quintiles (mean 12 869·8 patients per quintile; maximum absolute difference 0·9 per cent between volume groups). Some 550 hospitals (52·6 per cent) were grouped into the very low quintile. The number of hospitals declined across the different volume groups, with 101 and 61 hospitals in the high and very high‐volume categories respectively (*Table* 1 and *Fig*. 1 *a*). Mean patient age decreased steadily, from 70·3 years in very low‐volume hospitals to 66·6 years in the very high‐volume category (*P* < 0·001). This pattern was also found for the co‐morbidity score (*P* < 0·001) and mean LOS (21·7 *versus* 19·7 days respectively; both *P* < 0·001) (*Table* 1). Patients needing emergency surgery were treated more often in low‐volume than in high‐volume centres, accounting for 22·1 per cent of all operations in the lowest volume category compared with 15·3 per cent in very high‐volume hospitals (*P* < 0·001) (*Table* 2).

**Table 2:** Characteristics of patients undergoing colonic resection for rectal cancer in 2012–2015, according to hospital volume quintile

	Hospital volume quintile					
	Very low (*n* = 12 864)	Low (*n* = 12 738)	Medium (*n* = 12 989)	High (*n* = 12 916)	Very high (*n* = 12 842)	*P* *
**Ventilation > 48 h**	695 (5·4)	655 (5·1)	577 (4·4)	534 (4·1)	536 (4·2)	< 0·001
Mortality	250 (36·0)	224 (34·2)	200 (34·7)	192 (36·0)	155 (28·9)	0·080
**Emergency procedure**	2848 (22·1)	2617 (20·5)	2407 (18·5)	1992 (15·4)	1962 (15·3)	0·001†
Mortality	257 (9·0)	192 (7·3)	162 (6·7)	151 (7·6)	94 (4·8)	0·001
**Transfusion of ≥ 6 erythrocyte concentrates**	738 (5·7)	725 (5·7)	699 (5·4)	671 (5·2)	676 (5·3)	0·190
Mortality	169 (22·9)	169 (23·3)	151 (21·6)	167 (24·9)	130 (19·2)	0·140
**Stroke**	58 (0·5)	44 (0·3)	52 (0·4)	46 (0·4)	37 (0·3)	0·260
Mortality	12 (21)	16 (36)	16 (31)	9 (20)	9 (24)	0·290
**Pulmonary embolism**	97 (0·8)	118 (0·9)	91 (0·7)	112 (0·9)	96 (0·7)	0·220
Mortality	37 (38)	33 (28·0)	26 (29)	27 (24·1)	15 (16)	0·010
**Peritonitis/sepsis**	1424 (11·1)	1417 (11·1)	1269 (9·8)	1227 (9·5)	1193 (9·3)	< 0·001
Mortality	307 (21·6)	278 (19·6)	223 (17·6)	210 (17·1)	182 (15·3)	< 0·001
**Myocardial infarction**	112 (0·9)	103 (0·8)	113 (0·9)	100 (0·8)	93 (0·7)	0·640
Mortality	31 (27·7)	30 (29·1)	26 (23·0)	24 (24)	24 (26)	0·840
**Anastomotic leak**	1111 of 10 316 (10·8)	1156 of 10 052 (11·5)	1276 of 10 185 (12·5)	1253 of 10 108 (12·4)	1202 of 10 170 (11·8)	0·003
Mortality	121 (10·9)	105 (9·1)	106 (8·3)	84 (6·7)	76 (6·3)	< 0·001
**Relaparotomy, adhesiolysis or decompression**	760 (5·9)	745 (5·8)	862 (6·6)	827 (6·4)	884 (6·9)	0·001
Mortality	146 (19·2)	139 (18·7)	128 (14·8)	128 (15·5)	107 (12·1)	< 0·001
**(Protective) stoma**	4677 (36·4)	5300 (41·6)	5660 (43·6)	5968 (46·2)	6230 (48·5)	< 0·001
Mortality	222 (4·7)	199 (3·8)	152 (2·7)	163 (2·7)	127 (2·0)	< 0·001

Values in parentheses are percentages.

* χ^2^ test for difference between subgroups, except

† non‐parametric test for trend.

### In‐hospital mortality across hospital volume categories

A mean of 5·8 patients were treated annually in very low‐volume hospitals, whereas very high‐volume hospitals performed 52·6 rectal or rectosigmoid cancer resections for rectal cancer per year. There was a significant inverse association between hospital volume and mortality during hospital stay. The crude in‐house mortality rate ranged from 5·3 per cent in hospitals in the lowest volume category to 2·6 per cent in the highest‐volume centres (*P* < 0·001) (*Table* 1).

Similarly, after stratification for cancer location, very low‐volume hospitals had significantly higher inpatient mortality than very high‐volume centres (rectosigmoid cancer: 5·7 *versus* 2·8 per cent respectively, *P* = 0·001; rectal cancer: 5·3 *versus* 2·6 per cent, *P* < 0·001) (*Table* 1).

In a crude analysis, sex, age category, co‐morbidity and emergency procedures were significantly associated with both in‐hospital mortality and hospital volume category (*Table* 3). They were therefore considered potential confounders and included in the regression analysis.

**Table 3:** Crude odds ratios to determine factors influencing in‐house mortality

	Crude odds ratio	*P*
**Hospital volume quintile**		
Very low	1·00 (reference)	–
Low	0·82 (0·73, 0·92)	0·001
Medium	0·68 (0·60, 0·76)	< 0·001
High	0·63 (0·56, 0·71)	< 0·001
Very high	0·48 (0·42, 0·55)	< 0·001
**Sex**		
F	1·00 (reference)	
M	1·12 (1·03, 1·22)	0·008
**Age (years)**		
≤ 59	1·00 (reference)	
60–74	3·26 (2·80, 3·94)	< 0·001
≥ 75	9·54 (7·95, 11·45)	< 0·001
**Co‐morbidity score**	1·27 (1·26, 1·28)	< 0·001
**Emergency procedure**		
No	1·00 (reference)	
Yes	2·41 (2·21, 2·62)	< 0·001

Values in parentheses are 95 per cent confidence intervals.

In multivariable regression analysis, accounting for patient clustering within institutions and the effect of confounding variables, a highly significant decrease was found in hospital mortality following rectal cancer surgery across hospital volume categories. The adjusted OR for death was 42 per cent lower in very high‐volume centres and 22 per cent lower in both medium‐ and high‐volume centres compared with that in very‐low volume hospitals. In the multivariable model, the observed decrease in OR for in‐hospital death between the highest‐volume centres and the baseline rate was highly significant (*P* < 0·001), whereas the other volume categories had *P* values between 0·005 and 0·010 (*Table* 4).

**Table 4:** Logistic regression analysis of in‐hospital mortality by volume category, including hospital as random effect

	Adjusted odds ratio	*P*
**Hospital volume quintile**		
Very low	1·00 (reference)	
Low	0·80 (0·68, 0·95)	0·010
Medium	0·78 (0·65, 0·93)	0·005
High	0·78 (0·65, 0·94)	0·010
Very high	0·58 (0·47, 0·73)	< 0·001
**Sex**		
F	1·00 (reference)	
M	0·95 (0·86, 1·05)	0·330
**Age (years)**		
≤ 59	1·00 (reference)	
60–74	2·45 (1·99, 3·01)	< 0·001
≥ 75	4·80 (3·94, 5·86)	< 0·001
**Co‐morbidity score**	1·27 (1·26, 1·28)	< 0·001
**Emergency procedure**		
No	1·00 (reference)	
Yes	1·53 (1·38, 1·70)	< 0·001

Values in parentheses are 95 per cent confidence intervals.

When the number of patients was considered as a continuous variable, the regression model performed equally well, showing a highly significant linear trend between the number of patients treated and the risk of inpatient death after rectal cancer surgery (*Fig*. 1 *b* and *Table* 4).

As there was a difference in the number of emergency procedures between the hospital quintiles, a subgroup analysis was conducted, excluding all emergency cases but still accounting for all identified confounders. This analysis gave the same results, with a significant decrease in hospital mortality in high‐volume centres (*Fig*. 1 *c*; *Table* *S2*, supporting information).

### Complications and their management according to hospital volume

Anastomotic leak occurred more often in the medium‐ and high‐volume centres, with a rate of 12·5 per cent in medium‐volume hospitals. Prolonged ventilation (for more than 48 h) was less frequent in very high‐volume centres than in hospitals of the lowest volume category (4·2 *versus* 5·4 per cent; *P* < 0·001) (*Table* 2). No pattern was observed between hospital volume categories for transfusion of six or more erythrocyte concentrates, nor was there a trend for the rate of relaparotomy, adhesiolysis or surgical decompression and hospital volume categories. The incidence of peritonitis and/or sepsis as a secondary diagnosis was more frequent in the two lower‐volume hospital categories and then decreased steadily with increasing hospital volume (both 11·1 per cent *versus* 9·3 per cent in the highest‐volume category; *P* < 0·001). The incidence of pulmonary embolism did not significantly differ between hospital categories, nor did rates of stroke or myocardial infarction (*Table* 2).

**Figure 2:**
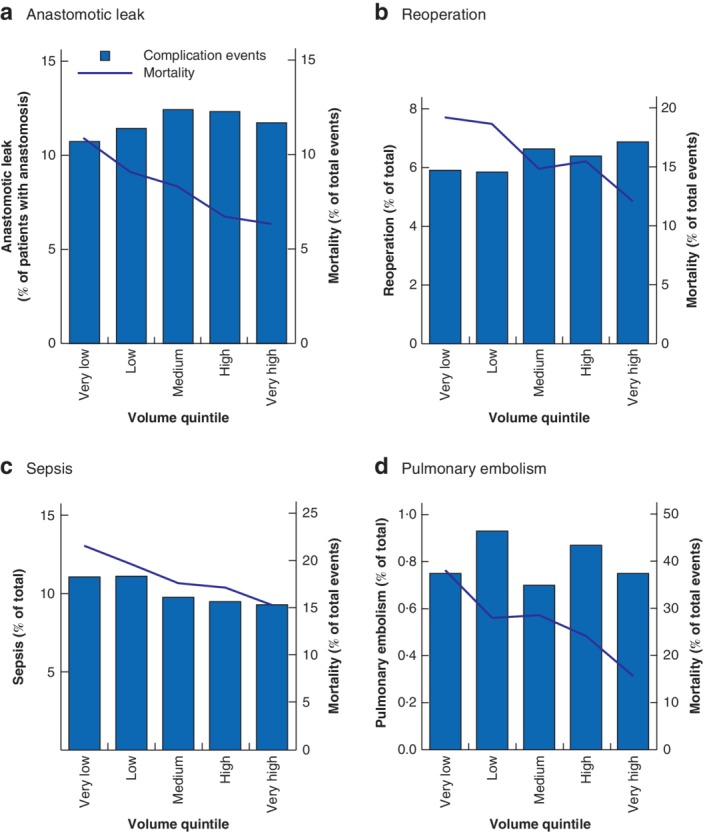
Postoperative complications and observed mortality for the complication according to hospital volume quintiles **a** Anastomotic leak, **b** reoperation, **c** sepsis and **d** pulmonary embolism events.

Although anastomotic leak was more common in higher‐volume hospitals, mortality rates in patients with anastomotic leak decreased with increasing hospital volume, ranging from 10·9 per cent in hospitals with the lowest caseload to 6·3 per cent in the highest‐volume centres (*P* < 0·001) (*Fig*. 2 *a* and *Tables* 2 and 5). Patients with a secondary diagnosis of peritonitis or sepsis had higher in‐hospital mortality when treated in very low‐volume centres than those treated in hospitals of the highest volume category (21·6 *versus* 15·3 per cent respectively; *P* < 0·001) (*Fig*. 2 *c* and *Tables* 2 and 5). Although a significant association between rates of relaparotomy, adhesiolysis or surgical decompression and hospital volume was not found, failure to rescue patients with one of these procedures was significantly lower in high‐volume than in low‐volume hospitals (mortality rate 12·1 per cent for very high‐volume centres *versus* 19·2 per cent for very low‐volume centres; *P* < 0·001) (*Fig*. 2 *b* and *Tables* 2 and 5). The mortality rate in patients with pulmonary embolism was reduced by over 50 per cent in very high‐volume centres compared with very low‐volume centres (15·6 *versus* 38 per cent respectively; *P* = 0·010) (*Fig*. 2 *d* and *Tables* 2 and 5).

**Table 5:** Complications and failure to rescue in lowest and highest volume quintiles

	Observed occurrence (%)				Observed mortality for the complication (%)			
	Overall occurrence	Very low volume	Very high volume	*P* *	Overall mortality	Very low volume	Very high volume	*P* *
Anastomotic leak	5998	10·8	11·8	0·003	492 (8·2)	10·9	6·3	< 0·001
Ventilation > 48 h	2997	5·4	4·2	< 0·001	1021 (34·1)	36·0	28·9	0·080
Transfusion of ≥ 6 erythrocyte concentrates	3509	5·7	5·3	0·190	786 (22·4)	22·9	19·1	0·140
Stroke	237	0·5	0·3	0·260	62 (26·2)	21	24	0·290
Pulmonary embolism	514	0·8	0·7	0·220	138 (26·8)	38	16	0·010
Peritonitis/sepsis	6530	11·1	9·3	< 0·001	1200 (18·4)	21·6	15·3	< 0·001
Myocardial infarction	523	0·9	0·7	0·640	135 (25·9)	27·7	26	0·840
Relaparotomy, adhesiolysis or decompression	4078	5·9	6·9	0·001	648 (15·9)	19·2	12·1	< 0·001

Values in parentheses are percentages.

* χ^2^ test for difference between subgroups (across all volume categories).

There were relatively more emergency admissions for rectal cancer in low‐volume categories, but these patients had a significantly lower mortality rate when admitted to a high‐volume centre (9·0 per cent in the lowest volume category *versus* 4·8 per cent in the highest volume category; *P* < 0·001) (*Table* 2).

## Discussion

This nationwide analysis has shown a significant and strong correlation between hospital volume and in‐hospital mortality for patients with rectal cancer in Germany. In very high‐volume centres with approximately 53 operations performed annually for rectal carcinoma, the adjusted OR for in‐hospital mortality was 0·58 compared with mortality in very low‐volume hospitals that perform only six operations for rectal carcinoma each year. This difference in mortality was found in both the unadjusted analysis and when adjusted for known confounders such as age, sex and emergency procedures. Furthermore, it displayed a nearly linear correlation with the annual caseload for each hospital. In addition, the postoperative complication rate did not correlate with hospital volume, although there were significantly increased rates of failure to rescue in low‐volume hospitals after both surgical (anastomotic leak and peritonitis) and non‐surgical (such as pulmonary embolism) complications.

Some 18·4 per cent of all operations were emergency procedures, an unexpectedly high proportion^6^, ^12^. These cases will increase the expertise of surgeons in individual hospitals, but could have biased the mortality analysis as they were not equally distributed across the quintiles. However, in a subgroup analysis that excluded emergency cases the same significant trend towards decreased mortality with higher‐volume quintiles was observed.

The mortality rate of 3·9 per cent in this study matches the 3·5 per cent rate found in a French nationwide analysis^13^. This French study also showed a clear correlation between in‐hospital mortality and the annual hospital caseload.

Several European countries, such as the UK and the Netherlands, have established protocols that centralize rectal cancer surgery. For example, over the last decade the training and centralization efforts made by the Dutch Colorectal Cancer Audit have led to a reduced 30‐day mortality rate, especially in patients with advanced tumour stages^14^, ^15^. As well as the positive impact on short‐term outcome, oncological parameters such as a negative circumferential resection margin and long‐term survival have improved within the Audit^16^, ^17^. In the UK, the Calman–Hine Report recommended similar strategic improvements to cancer services^18^. The subsequent centralization and specialization improved the short‐ and long‐term outcomes of affected patients and narrowed the gap between patients with rectal cancer in the UK and those in continental Europe^19^, ^20^, ^21^. Similar observations have been made for several other centralization programmes^22^, ^23^. The proportion of patients in the present study who had a laparoscopic resection was low (29·3 per cent) compared with that in the UK, where the rate is over 50 per cent, indicating that centralization and specialization also improves surgical approaches. This is also shown by the increased percentage of laparoscopic resection in higher‐volume hospitals.

In the present study, hospitals treating very few patients appeared to have increased mortality rates owing to high rates of failure to rescue. Recent analyses^24^, ^25^, ^26^ from Germany have also highlighted that the annual caseload for complex pancreas and oesophagus resections determines the long‐term survival and failure to rescue rate in these patients. Failure to rescue depends on additional factors apart from hospital volume, such as surgical experience and the availability of interventional radiologists, an endoscopy unit and an ICU. These structural requirements are found mainly in high‐volume centres and may account for the differences in postoperative outcomes after complex surgery^27^. Data from the American College of Surgeons National Surgical Quality Improvement Program^28^ and Medicare^29^ on postoperative mortality rates have shown that failure to rescue, rather than overall mortality, is strongly dependent on hospital volume. A subsequent analysis^30^ found that it was mainly hospital status (academic *versus* non‐academic), ICU capacity and academic character that determined the failure‐to‐rescue rate. A study^31^ focusing on failure to rescue after colorectal resection in the Netherlands demonstrated that low‐level ICU care in particular was associated with increased failure‐to‐rescue rates.

The main strength of this study is the sample size and completeness of data, and the adjustment for mortality and co‐morbidity^10^.

A major limitation of this analysis is the missing information on the influence of the individual surgeon and individual surgeons' expertise on the postoperative outcome. Furthermore, information on tumour stage and long‐term survival of patients was not available. Another limitation is the missing readmission data, as the statistics include only individual cases per hospital and readmission is not taken into account.

In view of the strong correlation found in this study between annual hospital caseload and postoperative morbidity and mortality following resection of rectal cancer, the introduction of highly specialized centres for rectal surgery is highly advocated to improve perioperative patient outcome. Board certification for specialized cancer centres by the German Cancer Society would be a first step in improving the quality of treatment, but great economic, political and social effort is needed to achieve this.

## Supporting information


**Table S1.** ICD‐10 German Modification codes used to calculate the co‐morbidity score (according to Stausberg and Hagn^10^)
**Table S2.** Logistic regression analysis of in‐hospital mortality by volume category including hospital as random effect (non‐emergency cases only)
