# Supplementary material for: Nationwide in‐hospital mortality rate following rectal resection for rectal cancer according to annual hospital volume in Germany
Source: BJS Open. 2020 Jan 10;4(2):310–9. doi: 10.1002/bjs5.50254 (PMC7093786; doi:10.1002/bjs5.50254)
Supplement: Supplementary file 1 — Table S1. ICD‐10 German Modification codes used to calculate the co‐morbidity score (according to Stausberg and Hagn10) Table S2. Logistic regression analysis of in‐hospital mortality by volume category including hospital as random effect (non‐emergency cases only) [file BJS5-4-310-s001.docx]

**BJS5_50254**

**Nationwide in-hospital mortality rate following rectal resection for rectal cancer according to annual hospital volume in Germany**

**J. Diers, J. Wagner, P. Baum, S. Lichthardt, C. Kastner, N. Matthes, H. Matthes, C.-T. Germer, S. Löb and A. Wiegering**

**Table S1** ICD-10 German Modification codes used to calculate the co-morbidity score (according to Stausberg and Hagn^10^)

| **ICD-10 GM codes** |
| --- |
| D10-D36, D60-D64, D65-D69  E00-E07, E50-E64, E65-E68  F10-F19, F30-F39  G10-G14, G90-G99  H53-H54  I10-I15, I26-I28, I30-I52, I60-I69, I70-I79, I80-I89  J09-J18, J30-J39, J60-J70, J80-J84, J90-J94, J95-J99  K35-K38, K40-K46, K55-K64, K65-K67, K70-K77  L80-L99  M00-M25, M40-M54  N17-N19, N25-N29, N30-N39  Q35-Q37  R10-R19, R30-R39, R40-R46, R50-R69  T15-T19, T80-T88 |

**Table S2** Logistic regression analysis of in-hospital mortality by volume category including hospital as random effect (non-emergency cases only)

|  | **adjusted odds ratio** | **95% confidence interval** | | **p-value** |
| --- | --- | --- | --- | --- |
| Case-load quintile  I  II  III  IV  V | 1.00  0.82  0.77  0.74  0.60 | -  0.68  0.62  0.59  0.47 | -  1.0  0.95  0.92  0.77 | -  0.051  0.013  0.007  p<0.001 |
| Sex  female  male | 1.00  1.00 | -  0.88 | -  1.13 | -  0.95 |
| Age category (yrs.)  ≤59  60 – 74  ≥75 | 1.00  2.34  4.77 | -  1.84  3.76 | -  2.99  6.05 | -  p<0.001  p<0.001 |
| Comorbidity score | 1.28 | 1.27 | 1.30 | p<0.001 |
